# Estrogen Receptor Alpha (ESR1) Gene Polymorphisms in Pre-eclamptic Saudi Patients

**DOI:** 10.12669/pjms.314.7541

**Published:** 2015

**Authors:** Hesham A. El-Beshbishy, Manal A. Tawfeek, Nevin M. Al-Azhary, Reham A. Mariah, Fawzia A. Habib, Lamya Aljayar, Abrar F. Alahmadi

**Affiliations:** 1 Hesham A. El-Beshbishy, Center for Genetics and Inherited Diseases, Taibah University, Madina, Saudi Arabia. Medical Laboratories Technology Department, Faculty of Applied Medical Sciences, Taibah University, Madina, Saudi Arabia. Biochemistry Department, Al-Azhar University, Nasr City, Cairo 11751, Egypt; 2 Manal A. Tawfeek, Clinical Pathology Department, Faculty of Medicine, Tanta University, Tanta, Egypt. Biochemistry & Molecular Medicine Department, College of Medicine, Taibah University, Madina, Saudi Arabia; 3 Nevin M. Al-Azhary, Clinical Pathology Department, National Cancer Institute, Cairo University, Cairo, Egypt. Biochemistry & Molecular Medicine Department, College of Medicine, Taibah University, Madina, Saudi Arabia; 4 Reham A. Mariah, Medical Biochemistry Department, Faculty of Medicine, Tanta University, Tanta, Egypt. Biochemistry & Molecular Medicine Department, College of Medicine, Taibah University, Madina, Saudi Arabia; 5 Fawzia A. Habib, Obstetrics and Gynecology Department, College of Medicine, Taibah University, Madina, Saudi Arabia; 6 Lamya Aljayar, Obstetrics and Gynecology Department, Madina Maternity Children Hospital, Madina, Saudi Arabia; 7 Abrar F. Alahmadi, Obstetrics and Gynecology Department, Madina Maternity Children Hospital, Madina, Saudi Arabia

**Keywords:** Pre-eclampsia, Pregnancy, Estrogen receptor alpha, Polymorphism

## Abstract

**Objectives:**

Pre-eclampsia causes maternal mortality worldwide. Estrogen receptor alpha (ESR1) gene polymorphisms were responsible for cardiovascular diseases. This case control study was conducted to investigate whether 2 polymorphic genes of ESR1 are associated with pre-eclampsia among Saudi women in Madina city, Saudi Arabia.

**Methods:**

Blood samples from 97 pre-eclamptic and 94 healthy pregnant women were analyzed using restriction fragment length polymorphism-polymerase chain reaction method. All the subjects were recruited randomly from outpatient clinics of Madina Maternity Children Hospital (MMCH), Madina, Saudi Arabia, between Dec. 2012 and Jan. 2014.

**Results:**

There was no association between pre-eclampsia and *PvuII* and *XbaI* ESR1 gene polymorphisms individually. TT/AA and TT/AG genotype combination existed significantly in pre-eclamptic patients compared to control. The frequency of *PvuII* and *XbaI* combined TT/AA genotypes between pre-eclamptic women was 36.1% *vs* 9.6%, however, frequency of *PvuII* and *XbaI* combined TT/AG genotypes between pre-eclamptic women was 3.1% *vs* 17%, compared to control. The homozygous T-A haplotype carriers showed high pre-eclampsia risk, independent of pregnancy, BMI and smoking status (adjusted odds ratio (OR): 3.26, 95% confidence interval (CI):1.71-9.21). The heterozygous T-A haplotype carriers did not differ from that of non-carriers (adjusted OR: 1.12, 95% CI: 0.47-2.75). No association was observed between pre-eclampsia and T-G, C-G and C-A haplotype of *PvuII* and *XbaIESR1* gene polymorphisms.

**Conclusions:**

T-A haplotype of homozygous associated with pre eclampsia not heterozygous carriers of ESR 1 *PvuII* and *XbaI* gene polymorphisms elicited high risk of pre-eclampsia. GG genotype of *XbaI* polymorphism decreased pre-eclampsia risk. Further studies using larger sample size are recommended to investigate the ESR 1 gene polymorphisms associated with pre-eclampsia.

## INTRODUCTION

Pre-eclampsia is a major medical illness affecting women after the 20^th^ week of gestation with an incidence of 2-5% worldwide, is characterized by elevated systemic vascular resistance, declined blood volume, abnormal renal hemodynamics and vascular endothelial cell disruption.^1^,^2^ Annually, up to 40.000 women could die from pre-eclampsia and eclampsia resulting from a shallow implanted placenta that leads to bad immune reaction involved with placental inflammatory mediators that acts on the vascular endothelium.^2^,^3^ Several mutations are associated with an increased risk of many complications.^4^,^5^ The M3T variant of the 235^th^ amino acid of angiotensinogen gene has significant correlation with hypertension and pre-eclampsia.^6^

Estrogen hormone influences several physiological processes, which include female reproduction, bone integrity and cardiovascular function.^7^ Estrogen receptor α (ESR1) functions as a ligand-activated transcription factor that located in nucleus and cytoplasm, forming an estrogen/ER complex.^8^ ESR1 (595 amino acids, molecular weight ~66 kDa) is translated from a 6.8Kb mRNA that contains 8 exons and derived from a gene located on long arm of chromosome 6q25.1.^9^ The N-terminus of ESR1 (encoded by exon 1 and exon 2) have major role in activation of ESR1 dependent genes and any mutations in this region have been linked with hypertension.^9^-^12^

ESR1 polymorphic genes have been studied in several illness as hypertension, coronary artery diseases and stroke.^13^-^16^ The ESR1 *PvuII* and *XbaI* polymorphisms homozygous T-A haplotype carriers elicited pre-eclampsia high risk.^8^ In addition, ESR1 *XbaI* polymorphism GG genotype was associated with low risk of fetal growth abnormality in pre-eclampsia. ESR1 polymorphisms was associated with vein thrombosis and also with spontaneous abortion.^17^,^18^ The more prominent single nucleotide ESR1 gene polymorphisms are c454-397T>C and c454A>G site which are located in first intron of ESR and consider most frequently studied polymorphisms of ESR1 gene (ESR1 c.454 −397T>C: *PvuII* restriction site [rs2234693] and c.454 −351A>G: *XbaI* restriction site [rs9340799].^19^ Also, it was reported that, the common variant rs11646213 is associated with preeclampsia in Han Chinese women.^20^ A retrospective study carried out in 2003 involved women who delivered at King Fahd hospital (Jeddah, Saudi Arabia) in a 10-year period (1992–2001), revealed incidence of pre-eclampsia of 2.47%, with a high proportion among nulliparous women and those near the end of reproductive age. Severe maternal and neonatal complications were noticed in women with pre-eclampsia.^21^

This study aimed to investigate association between ESR1 gene polymorphisms and pre-eclampsia among Saudi women in Madina city, Saudi Arabia.

## METHODS

This study was approved by medical ethics committee of faculty of Applied Medical Sciences, Taibah University, Madina, Saudi Arabia. The recruited subjects agreed to give us their verbal informed consent instead of the written one for genetic analysis and we got the approval by the ethics committee. We documented the participant consents in our data excel sheet.

### Patient selection

Subjects were recruited randomly from Madina Maternity Children Hospital (MMCH) outpatient clinics, Madina, Saudi Arabia, between Dec. 2012 and Jan. 2014. All women involved in this study were Saudi lived in Madina. Study group involved 94 healthy normotensive pregnant women (systolic blood pressure <130 mmHg and diastolic blood pressure <85 mmHg) with uncomplicated pregnancy and 97 pre-eclamptic patients. Pre-eclampsia was associated with high blood pressure (>160 mmHg systolic or >110 mmHg diastolic) that occurred after 20^th^ week of gestation in a woman with previously normal blood pressure and characterized with proteinuria (>5g/day) (ACOG, 2002). Blood pressure backed to normalcy by 12-week postpartum. Fetal growth restriction was diagnosed in case of fetal birth weight was under the 10^th^ percentile for gestational age.^22^

### Exclusion criteria

Patients with multifetal gestation, Diabetes mellitus, autoimmune disease malignancy, renal diseases, liver insufficiency and hypertension were excluded.

### Sampling

Venous blood samples were collected from all the participants and divided into two aliquots. One was used for measurements of biochemical parameters and the other was placed in EDTA-containing vacutainers for genomic DNA extraction. The measured parameters included age (years), presence of smoking, body mass index (BMI), gestational age and blood pressure mm/Hg (systolic and diastolic). The measured biochemical parameters included serum urea (mg%), serum creatinine (mg%), serum triglycerides (mg%), serum HDL-cholesterol (mg%) and serum LDL-cholesterol (mg%). Biochemical kits were obtained from Randox (UK).

### DNA extraction

Genomic DNA was extracted from blood samples using QIAamp DNA blood mini kit (Qiagen, Germany) according to the manufacturer instructions. The extracted DNA quantified by measuring its absorbance at 260 nm using GeneQuest (model CE2301, USA) as absorbance 1 = 50ng/ml. The DNA was quantified on 1% agarose gel electrophoresis stained with ethidium bromide.

### ESR1 gene polymorphisms

Restriction fragment length polymorphism polymerase chain reaction (RFLP-PCR) was used to detect ESR1 gene. Existence of ESR1 intron 1-397T>C (*PvuII* or IVS1-397) and −351A>G (*XbaI* or IVS1−351) genotypes were investigated. A PCR product(255 bp) was obtained using forward primer (5′-CAGGGTTATGTGGCAATGAC-3′) and reverse primer (5′-TACCTATAAAAATGACAAAATGAAAT-3′) in 25 Ml reaction mixture containing PCR buffer, 1.5mmol/L MgCl_2_, 0.25mmol/L dNTPs mix, 0.4 Mmol/L of each primer, 1.5 U *Taq* DNA polymerase and ~100 ng DNA.^23^ The PCR conditions included denaturation at 94°C for 4 minutes followed by 35 cycles including denaturation at 94°C for 30 second, annealing at 60°C for 30 second and extension at 72°C for 30 second, ending with extension at 72°C for 10 min using a thermal cycler (Eppendorff`s, Germany).

The PCR product contained a part of intron 1 of ESR1 gene, was digested with *PvuII* and *XbaI* enzymes at 37°C for 10 hrs, to produce a 255 bp fragment which comprises the allele (C) or 97+158 bp fragments which comprises the allele (T) and of 255 bp for the allele (G) or 142+113 bpfor the allele (A), respectively. Results described as AA, GG or AG for *Xba I* and CC, TT or CT for heterozygous allele for *PvuII*. The products were electrophoresed on 3% agarose gel and stained with ethidium bromide to visualize DNA under ultraviolet illumination.

### Statistical analysis

Statistical analysis was done using GraphPad Prism 5 software (GraphPad, SD, USA). Data obtained from pre-eclamptic and control groups were compared using Student`s t-test. Fisher’s exact test was then used to compare polymorphisms distribution in codons 10 and 87 of exon 1 of ESR 1 gene in the two studied groups. Data are reported as median (25–75 percentile) for continuous variables and number (%) for categorical variables. Data were expressed as mean±standard error (SE). The *p*<0.05 was considered statistically significant.

## RESULTS

A total of 191 samples were included in this study. These samples comprised 97 pre-eclamptic women as patients group and 94 normal pregnant women as control group. The characteristics of study subjects; pre-eclamptic patients who did not receive antihypertensive medication and healthy pregnant women enrolled in this study were described in Table-I.

**Table-I:** Clinical and biochemical characteristics of the studied groups.

		Controls (n=94)	Pre-eclamptic patients (n=97)
^a^Age (years)	Range	19-30	18-31
		24±2.9	24±2.1
^b^Gestational age at delivery (weeks)	Range	38-41	32-35
		38±2.4	31±1.8*
Smokers (%)		8 (8.5%)	10 (10.64%)
Fetal birth weight (g)		3451±454	1491±521*
Systolic blood pressure (mm/Hg)		119±7.0	190±14*
Diastolic blood pressure (mm/Hg)		78±5.0	113±10*
BMI (Kg/m^2^)		21.1±3.8	22.7±3.1*
Urea (mg%)	Range	15-35	45-50
		18.2±2.45	39.8±2.9*
Creatinine (mg%)	Range	0.5-1.0	1.0-1.1
		0.48±0.004	0.61±0.005*
Triglycerides (mg%)	Range	161-170	170-173
		116±15.2	156±17.5
HDL-C (mg%)	Range	35-40	41-45
		36±1.7	43±2.6
LDL-C (mg%)	LDL-C (mg%) Range	111-121	116-126
		101±3.7	107±2.8

Data were expressed as mean±SE for continuous parametric variables.

a,b Data were expressed as median (25-75 percentile) for continuous non-parametric variables Data were expressed as number (%) for biochemical parameters, BMI: body mass index.

* Statistical significance compared to the control group, *P*<0.05.

There were no significant differences between demographic profile of control and pre-eclamptic subjects. Systolic and diastolic blood pressures, serum urea and serum creatinine were significantly elevated in pre-eclamptic women compared to control. Maternal age showed no significant difference among pre-eclamptic women, however, BMI was significantly higher in pre-eclamptic group. Gestational age at delivery was significantly lower in pre-eclamptic patients, which explains low fetal birth weight in pre-eclamptic patients (Table-I).

### ESR1 genotypes and allele frequencies

The genotype and allele frequencies of *PvuII* and *XbaI* ESR1 gene polymorphism in pre-eclamptic and control groups are presented in Table-II. The *PvuII* and *XbaI* ESR1 polymorphism haplotype frequencies are presented in Table-III. There was no significant differences between the pre-eclamptic women regarding genotyping and allele frequencies of *PvuII* and *XbaI* polymorphism, however, the TT/AA and TT/AG genotype combination existed significantly in pre-eclamptic patients compared to control. Frequency of *PvuII* and *XbaI* combined TT/AA genotypes between pre-eclamptic women was 36.1% *versus* 9.6%, however, frequency of *PvuII* and *XbaI* combined TT/AG genotypes between pre-eclamptic women was 3.1% *versus* 17%, compared to control.

**Table-II:** The genotypes distribution and alleles frequencies of PvuII and XbaI ESR 1 gene polymorphisms in women with pre-eclampsia compared to normotensive healthy pregnant women.

	Controls (n=94)	Pre-eclamptic patients (n=97)
*PvuII genotypes frequencies*		
TT	30 (31.9%)	24 (24.7%)
TC	44 (46.8%)	52 (53.6%)
CC	20 (21.3%)	21 (21.6%)
	X^2^=0.61, d.f.=2, p>0.05	
*PvuII alleles frequencies*		
T	110 (58.5%)	106 (54.6%)
C	78 (41.5%)	88 (45.4%)
*XbaI genotypes frequencies*		
AA	26 (27.7%)	30 (30.9%)
AG	58 (61.7%)	54 (55.7%)
GG	10 (10.6%)	13 (13.4%)
	X^2^=0.23, d.f.=2, p>0.05	
*XbaI alleles frequencies*		
A	104 (55.3%)	117 (60.3%)
G	84 (44.7%)	77 (39.7%)

**Table-III:** Haplotype frequencies of PvuII and XbaI polymorphisms in the pre-eclamptic patients and control group.

	Controls (n=94)	Pre-eclamptic patients (n=97)
TT/AA	9 (9.6%)	35 (36.1%)*
TT/AG	16 (17%)	3 (3.1%)*
TT/GG	3 (3.2%)	2 (2.1%)
TC/AA	11 (11.7%)	3 (3.1%)
TC/AG	34 (36.2%)	39 (40.2%)
TC/GG	4 (4.3%)	1 (1%)
CC/AA	3 (3.2%)	2 (2.1%)
CC/AG	8 (8.5%)	4 (4.1%)
CC/GG	6 (6.4%)	8 (8.2%)

* significant difference compared to control, P<0.05.

The homozygous T-A haplotype carriers showed increased pre-eclampsia risk, independent of pregnancy, BMI and smoking status (adjusted odds ratio (OR): 3.26, 95% confidence interval (CI):1.71-9.21, Table-IV). The risk of heterozygous T-A haplotype carriers not different from that of non-carriers (adjusted OR: 1.12, 95% CI: 0.47-2.75). No association was noticed between pre-eclampsia and T-G, C-G and C-A haplotype of the ESR1 *PvuII* and *XbaI* polymorphism after adjustment pregnancy BMI and smoking status (Table-IV).

**Table-IV:** T-A Haplotype carriers and non-carriers of the PvuII and XbaI ESR1 gene polymorphisms in the pre-eclamptic patients and control group.

	Controls (n=94)	Pre-eclamptic patients (n=97)	Adjusted odds ratio (OR)
Homozygous T-A carriers	9 (9.6%)	35 (36.1%)*	Homozygous T-A carriers vs. heterozygous T-A carriers plus non-carriers, p=0.05*. Adjusted OR: 4.94, 95% CI: 1.18-13.17 homozygous T-A carriers vs non-carriers, p=0.05. ^#^Adjusted OR: 1.12, 95% CI: 0.47-2.75
Heterozygous T-A carriers	61 (64.9%)	45 (46.4%)*	Heterozygous T-A carriers vs. non-carriers, p>0.05
Non-carriers	24 (25.5%)	17 (17.5%)	

Adjustment was conducted for age, BMI and smoking status.

ESRα: estrogen receptor alpha; BMI: body mass index.

## DISCUSSION

Pre-eclampsia is a major cause of maternal mortality worldwide. The symptomatic treatment of pre-eclampsia that is available includes the premature termination of pregnancy. The genetic identification of high-risk women prior to development of pre-eclampsia is crucial.^2^

It was reported that, estrogen may influence the risk of pre-eclampsia *via* complex mechanisms mediated by ESR1 through alterations in coagulation and fibrinolysis pathways. Estrogen hormone tends to initiate thrombosis via decreasing total cholesterol and LDL cholesterol and elevating triglycerides serum levels.^13^,^24^,^25^ Estrogen level was increased during normal pregnancy as estrogen is highly produced by placenta and embryo during pregnancy. In spite of increment of estrogen levels during pregnancy; blood pressure kept normal.^26^,^27^ Low ESR1 molecules were noticed in pre-menopausal women suffered from coronary arteries atherosclerosis compared with those with normal arteries.^28^,^29^ ESR1 protect against vascular injury.^30^,^31^ As estrogen effects are mediated *via* estrogen receptors, variations in ESR could have an important role in pregnancy maintenance.^10^

In this study, we investigated association between 2 polymorphic genes of the ESR1 and pre-eclampsia in Saudi women located in Madina region. It was revealed that, reduced ESR1 expression as a result to ESR1 gene sequence variations can be responsible for vasoconstriction that initiate pre-eclampsia risk in pregnant women and subsequently abortion. ESR1 play a pivotal role in systemic circulation maintenance during pregnancy.^10^,^32^ In addition, ESR1 *PvuII* gene polymorphism was found to be associated with perinatal morbidity.^33^

A genetic haplotype is identified as a combination of sets of alleles on the same chromosomal segment that tend to be transmitted as a block.^34^ The haplotype analysis was used to analyze the association between haplotypes and pre-eclampsia to establish haplotypes of ESR1 gene polymorphisms. We found that, homozygous and heterozygous T-A haplotype carriers of ESR1 *PvuII* and *XbaI* genotypes, have increased risk of pre-eclampsia. In another study, among premature infants, the homozygotes for *PvuII* polymorphism have a higher risk of complications in perinatal period.^33^ No association was observed between pre-eclampsia and C-G, T-G and C-A haplotypes of ESR1 *PvuII* and *XbaI* polymorphisms. On other hand, CG genotype of the ESR1 *XbaI* polymorphism was correlated with low risk of pre-eclampsia.

In conclusion, homozygous associated with pre eclampsia not heterozygous T-A haplotype carriers of ESR 1 *PvuII* and *XbaI* gene polymorphisms elicited pre-eclampsia risk. Further studies using large sample size are required to gain insight and explore the mechanism of ESR 1 gene polymorphism in pre-eclampsia.

## References

[1] NHBPEP- National High Blood Pressure Education Program (1990). Working group report on high blood pressure in pregnancy: Consensus report. Am J Obstetr Gynecol.

[2] Roberts JM, Cooper DW (2001). Pathogenesis and genetics of pre-eclampsia. Lancet.

[3] Selim M, Elshmry N, Rashed E (2013). The role of novel biomarker in early prediction of preeclampsia in pregnant rats. J Blood Disord Transf.

[4] Thawnashom K, Tungtrongchitr R, Petmitr S, Pongpaew P, Phonrat B (2005). MTHFR polymorphism(C677T) in relation to homocysteine concentration in overweight and obese thais. Southeast Asian J Trop Med Public Health.

[5] Ibrahim ZM, Metawie MA, El-Baz AM, El-Bahie MA (2012). Methylenetetrahydrofolate C677T polymorphism and pre-eclamptic Egyptian women. Middle East Fert Soc J.

[6] Ward K, Hata A, Jeunemaitre X, Helin C, Nelson L, Namikawa C (1993). A molecular variant of angiotensinogen associated with preeclampsia. Nat Genetics.

[7] Araújo L, Dias de Rezende L, Souza L, Daltoe R, Madeira K, Ferreira Paes M (2001). Prevalence of estrogen receptor alpha PvuII (c454-397T>C) and XbaI (c454A>G): Polymorphisms in a population of Brazilian women. Brazilian Arch Biol Technol.

[8] Jakimiuk AJ, Malgorzata N, Bogusiewicz M, Adamiak A, Skorupski P, Miotla P (2007). Prevalence of estrogen receptor α PvuII and XbaI polymorphism in population of Polish postmenopausal women. Folia Histochemical Cytobiology.

[9] Parker MG (1993). Structure and function of the estrogen receptor. J Neuroendocrinol.

[10] Lehrer S, Sanchez M, Song HK, Dalton J, Levine E, Savoretti P (1990). Oestrogen receptor B-region polymorphism and spontaneous abortion in women with breast cancer. Lancet.

[11] Lehrer S, Rabin J, Kalir T, Schachter BS (1993). Estrogen receptor variant and hypertension in women. Hypertension.

[12] Smith EP, Boyd J, Frank GR, Takahashi H, Cohen RM, Specker B (1994). Estrogen resistance caused by a mutation in the estrogen-receptor gene in a man. N Engl J Med.

[13] Mendelsohn ME, Karas RH (1999). The protective effects of estrogen on the cardiovascular system. N Engl J Med.

[14] Gennari L, Merlotti D, De Paola V, Calabrò A, Becherini L (2005). Estrogen receptor gene polymorphisms and the genetic of osteoporosis: a huge review. Am J Epidemiol.

[15] Roberts JM, Gammill H (2005). Pre-eclampsia and cardiovascular disease in later life. Lancet.

[16] Rigo J, Boze T, Derzsy Z, Derzbach L, Treszl A, Sobel G (2006). Family history of early onset cardiovascular disorders is associated with a higher risk of severe preeclampsia. Euro J Obstetr Gynecol Reprod Biol.

[17] Aléssio AM, Höehr NF, Siqueira LH, Ozelo MC, Mansur AP (2007). Association between estrogen receptor alpha and beta gene polymorphisms and deep vein thrombosis. Thrombosis Res.

[18] Anousha A, Hossein-Nezhad A, Biramijamal F, Rahmani A, Maghbooli Z (2013). Association study of estrogen receptor alpha gene polymorphisms with spontaneous abortion: Is this a possible reason for unexplained spontaneous abortion?. Bio Med Res Int.

[19] Silva IV, Rezende LCD, Lanes SP, Souza LS, Madeira KP (2010). Evaluation of PvuII and XbaI polymorphisms in the estrogen receptor alpha gene (ESR1) in relation to menstrual cycle timing and reproductive parameters in post-menopausal women. Maturitas.

[20] Wan J-P, Zhao H, Li T, Li C-Z, Wang X-T, Chen Z-J (2013). The common variant rs11646213 is associated with preeclampsia in Han Chinese women. PLoS One.

[21] Al-Mulhim A, Abu-Heija A, Al-Jamma F, El-Harith E.-H.A (2003). Pre-eclampsia: maternal risk factors and perinatal outcome. Fetal Diag Ther.

[22] Joubert K (2000). Standards of the body mass and body length of birth in Hungary on the basis of the 1990-1996 nation-wide live born data. Magy Noorv Lapja.

[23] Molvarec A, Ver A, Fekete A, Rosta K, Derzbach L, Derzsy Z (2007). Association between estrogen receptor α(ESR1) gene polymorphisms and severe preeclampsia. Hypertension Res.

[24] Vandenbroucke JP, Koster T, Briet E, Reitsma PH, Bertina RM, Rosendaal F (1994). Increased risk of venous thrombosis in oral-contraceptive users who are carriers of factor V Leiden mutation. Lancet.

[25] Jick H, Derby LE, Myers MW, Vasilakis C, Newton KM (1996). Risk of hospital admission for idiopathic venous thromboembolism among users of postmenopausal oestrogens. Lancet.

[26] Oertel GW, West CD, Eik-Nes KB (1959). Isolation and identification of estrone, estradiol and estriol in human pregnancy plasma. J Clin Endocrinol Metab.

[27] Tamura M, Nakayama T, Sato I, Sato N, Izawa N (2008). Haplotype-based case-control study of estrogen receptor α (ESR1) gene and pregnancy-induced hypertension. Hypertension Res.

[28] Mendelsohn ME (2002). Protective effects of estrogen on the cardiovascular system. Am J Cardiol.

[29] Mishell DR, Mendelsohn ME (2002). Introduction: the role of hormone replacement therapy in prevention and treatment of cardiovascular disease in postmenopausal women. Am J Cardiol.

[30] Karas RH, Schulten H, Pare G, Aronovitz M, Ohlsson C, Gustafsson J-A (2001). Effects of estrogen on the vascular injury response in estrogen receptor alpha, beta (double) knockout mice. Circulation Res.

[31] Pare G, Krust A, Karas RH, Dupont S, Aronovitz M (2002). Estrogen receptor-alpha mediates the protective effects of estrogen against vascular injury. Circulation Res.

[32] Byers MJ, Zangel A, Phernetton TM, Lopez G, Chen DB (2005). Endothelial vasodilator production by ovine uterine and systemic arteries: ovarian steroid and pregnancy control of ER alpha and ER beta levels. J Physiol.

[33] Derzbach L, Treszl A, Balogh A, Vasarhelyi B, Tulassay T, Rigo J (2005). Gender dependent association between perinatal morbidity and estrogen receptor-alpha PvuII polymorphism. J Perinatal Med.

[34] Sundermann EE, Maki PM, Bishop JR (2010). A review of estrogen receptor αgene (ESR1) polymorphisms, mood, and cognition. Menopause.

